# APOBEC in breast cancer: a dual player in tumor evolution and therapeutic response

**DOI:** 10.3389/fmolb.2025.1604313

**Published:** 2025-04-28

**Authors:** Haiqi Lu, Zelin Lu, Yufei Wang, Miaoqin Chen, Guangliang Li, Xian Wang

**Affiliations:** ^1^ Department of Medical Oncology, Sir Run Run Shaw Hospital, Zhejiang University School of Medicine, Hangzhou, Zhejiang, China; ^2^ Department of Breast Medical Oncology, Zhejiang Cancer Hospital, Hangzhou, Zhejiang, China

**Keywords:** APOBEC mutagenesis, breast cancer subtypes, immune checkpoint blockade, tumor mutational burden, therapeutic resistance

## Abstract

The APOBEC (Apolipoprotein B mRNA-editing enzyme, catalytic polypeptide-like) family of cytidine deaminases has emerged as pivotal a contributor to genomic instability and adaptive immunity through DNA/RNA editing. Accumulating evidence underscores their dual role in breast carcinogenesis—driving tumor heterogeneity via mutagenesis while simultaneously shaping immunogenic landscapes. This review synthesizes current insights into APOBEC-mediated molecular mechanisms, focusing on their clinical implications across breast cancer subtypes. Notably, APOBEC-driven mutagenesis correlates with elevated tumor mutational burden (TMB), replication stress vulnerability, and immune checkpoint inhibitor (ICI) responsiveness. Paradoxically, these mutations also accelerate endocrine therapy resistance and subclonal diversification. We propose APOBEC mutational signatures as predictive biomarkers for ICI efficacy and discuss therapeutic strategies leveraging APOBEC activity, including ATR inhibition and hypermutagenic immunotherapy. Harnessing APOBEC’s duality—balancing its pro-immunogenic effects against genomic chaos—may redefine precision oncology in breast cancer.

## 1 Introduction

Breast cancer has become a major focus of attention as the most common type of cancer among women worldwide. In 2020, global cancer statistics showed that female breast cancer surpassed lung cancer as the most frequently diagnosed cancer, with approximately 2.3 million cases, accounting for 11.7% of all new cancers, and its incidence continues to rise (Sung et al., 2021). The clinical behavior and response to therapy in breast cancer are influenced by various factors, including pathology, genomic alterations, gene expression and tumor microenvironment characteristics. However, clinical parameters for guiding decision-making remain imperfect, especially for advanced breast cancers that develop drug resistance. Therefore, it is very crucial to understand tumor heterogeneity and genetic drivers (Nolan et al., 2023).

The APOBEC family comprises three major functional elements: a catalytic structural domain, a cofactor interaction region and a sequence element that determine the subcellular localization of the protein (Salter et al., 2016). The substrate-binding groove, formed by four loops (1, 3, 5, and 7) surrounding the active site, governs the function of most APOBEC enzymes, including substrate binding, dinucleotide preference and catalysis (Pecori et al., 2022; Kouno et al., 2017). APOBECs can modify RNA through base deamidation, resulting in sequence changes detectable by sequencing (Helm and Motorin, 2017). Additionally, the APOBEC family is capable of deaminating DNA, such as the original discovery that apolipoprotein B (apoB) mRNA contains a base modification from C to U that is not encoded by the genome (Chen et al., 1987; Powell et al., 1987). In conclusion, these studies highlight APOBECs as both DNA mutators and RNA editors (Pecori et al., 2022).

APOBEC1, the first characterized family member, is expressed in the gastrointestinal tract (in both mice and human) and immune cells (in mice only). Its only confirmed physiologic target is the apolipoprotein B (APOB) mRNA, though it can also deaminate DNA, a process linked to cancer (Niavarani et al., 2018; Saraconi et al., 2014; Rogozin et al., 2019). In constrast, APOBEC2 does not deaminate RNA or DNA but binds to DNA at specific promoter regions to act as a transcriptional repressor (Ohtsubo et al., 2017). APOBEC3 is best known for its role in innate immune protection against viral infections and for generating mutation in various human cancer cell types (Petljak et al., 2022a; Ng et al., 2019). It comprises seven sub-family members in humans (designated A3A to A3H), with A3A being a primary driver of mutations. *In vitro*, studies have shown that overexpression of A3B gene induces extensive C>T mutations and increases uracil levels in the genome (Burns et al., 2013a). In addition, A3B expression correlates with cell cycle regulation and DNA repair (Ng et al., 2019), restrains A3A-dependent mutagenesis and contributes its own smaller mutation burdens (Chen et al., 1987). APOBEC3A (A3A) and APOBEC3B (A3B) are recognized as key drivers of APOBEC3-mediated mutagenesis in cancer, inducing somatic mutations that contribute to disease progression and therapeutic resistance (Ng et al., 2019; Maciejowski et al., 2020; Cortez et al., 2019; Durfee et al., 2023; Naumann et al., 2023). Because of the above-mentioned specific functions of APOBEC3, it induces mutations that are prevalent in cancer (Durfee et al., 2023; Middlebrooks et al., 2016; Isozaki et al., 2023). APOBEC3G plays a crucial role in innate immunity and antiviral defense by introducing mutations into viral DNA through deamination during HIV infection, thereby inhibiting viral replication. However, HIV-1’s Vif protein counteracts this by recruiting an E3 ubiquitin ligase complex to degrade APOBEC3G. Genetic variations in Vif affect its ability to degrade APOBEC3G, reflecting HIV-1’s adaptability. For example, research in North India found that Vif B/C recombinants have an enhanced capacity to degrade APOBEC3G, highlighting the virus’s adaptive traits. These findings expand our understanding of APOBEC’s functions beyond tumorigenesis (Ronsard et al., 2015). APOBEC-associated mutational signatures are widespread in cancer, found in over 70% of cancer types and approximately 50% of all cancer genomes. Specifically, APOBEC is implicated in cancers such as bladder (Middlebrooks et al., 2016), lung (Durfee et al., 2023), prostate, esophageal, pancreatic, endometrial, renal cell carcinoma, and breast cancer, contributing to carcinogenesis, heterogeneity, and treatment resistance. In breast cancer, APOBEC contributes to immunogenic mutations, potentially enhancing response to immune checkpoint inhibitors in tumors with high mutation burdens (Isozaki et al., 2023). Furthermore, APOBEC3 mutagenesis is a frequent mediator of therapy resistance in breast cancer, including endocrine therapy, and may serve as a prognostic biomarker. These findings underscore the significant association between APOBEC activity and breast cancer development and treatment response.

**FIGURE 1:**
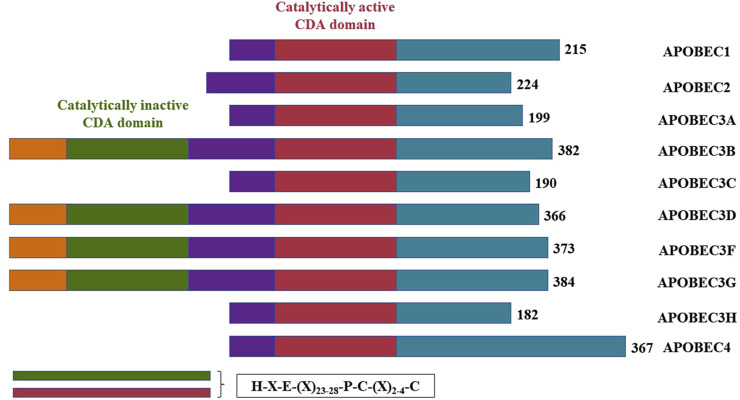
Gene structure of APOBEC family members.

In this review, we summarize the clinical implications and prognostic role of APOBEC in relation to its function in molecular biology for different breast cancer subtypes (Figure 1).

## 2 APOBEC and cancer

### 2.1 APOBEC promotes cancer evolution and heterogeneity

**FIGURE 2:**
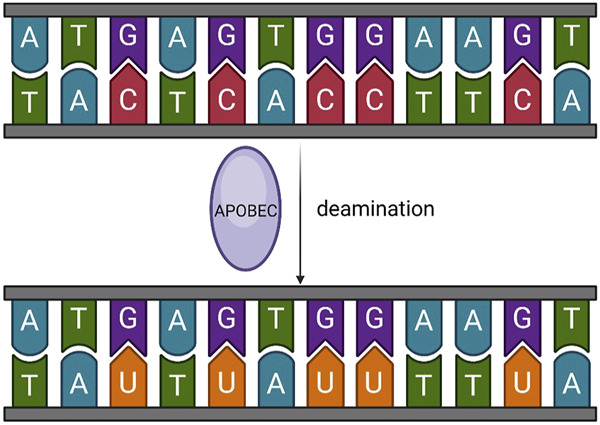
Basic function of APOBEC. APOBEC deaminates cytosine in DNA and RNA, leading to somatic mutations, DNA breaks, RNA modifications, or DNA demethylation.

Cancer arises DNA mutations, which lead to diverse changes ranging from single nucleotide alterations to chromosome rearrangement, fostering tumor diversity. The APOBEC family deaminates cytosine in DNA and RNA, leading to somatic mutations, DNA breaks, RNA modifications, or DNA demethylation (Figure 2). This activity serves as mutagenic fuel for cancer evolution and heterogeneity (Swanton et al., 2015), particularly in bladder and breast cancers (Middlebrooks et al., 2016; Swanton et al., 2015; Burns et al., 2013b; Alexandrov et al., 2013; Roberts et al., 2013).

Previous studies have identified over 30 SBSs (single-base substitutions) signatures in malignant tumors, with SBS2 and SBS13 attributed to APOBEC activity (Alexandrov et al., 2020). APOBEC3 may diversify extrachromosomal oncoproteins and promote the evolution of ecDNA-containing tumors by mutagenesis of ecDNA, exploiting its cyclic nature, and repeated mutations (Bergstrom et al., 2022). Notably, 59.2% of hypermutated breast cancers exhibit a dominant APOBEC mutational signature, while 36.4% show a dominant mismatch repair-deficient (dMMR) signature (Barroso-Sousa et al., 2020), which is a significant mechanism related to hyper-mutation. In particular, breast cancer patients with APOBEC-rich features exhibit higher mutational loads and APOBEC3B protein levels compared to other tumors (Krug et al., 2020), and A3B is also associated with a hypermutated phenotype (Roberts and Gordenin, 2014). Additionally, SBS2 and SBS13 mutations are also enriched in metastatic caners. Martínez-Jiménez et al. revealed that APOBEC mutation burden significantly increases in six metastatic tumor types (breast, colorectal, stomach, kidney, prostate and pancreatic neuroendocrine carcinomas) (Martínez-Jiménez et al., 2023). Compared to early breast cancer, HR+/HER2− metastatic breast cancer shows an increasing trend in SBS2 and SBS13 signatures. The APOBEC mutational signature S13 increases significantly during the transition from the luminal A subtype to the more aggressive luminal B and HER2-enriched subtypes (Park et al., 2023), correlating with poor outcome and resistance to endocrine therapy (Bertucci et al., 2019). APOBEC signatures are also observed in the late stages of breast cancer evolution (Nik-Zainal et al., 2016). Furthermore, most TMB-high breast metastatic invasive lobular carcinoma (mILCs) harbor an APOBEC trinucleotide signature (14/16; 88%) (Sokol et al., 2019). APOBEC activity varies across breast cancer subtypes, with typically higher activity observed in triple-negative breast cancer (TNBC), potentially contributing to its malignant transformation and progression (Petljak et al., 2022b). In conclusion, APOBEC is strongly associated with cancer occurrence and evolution.

### 2.2 APOBEC in breast cancer

Burns et al. quantified mRNA levels of APOBEC family members in 38 independent breast cancer cell lines and found that only A3B mRNA was upregulated in most cell lines (28/38) (Burns et al., 2013a). Furthermore, upregulation of A3B and high uracil load resulted in 3–5 times more mutations. In addition, inactivation of TP53 is a prerequisite for bypassing DNA damage checkpoints caused by A3B. Likewise, Periyasamy et al. investigated the regulation of A3B gene expression and illuminated negative correlation between A3B expression with p53 (protein expressed by TP53). It occurs through induction of p21 (CDKN1A) and recruitment of repressive DREAM complex to A3B gene promoter, so that mutation of p53 represses the recruitment and leads to upregulation of A3B (Periyasamy et al., 2017). On the other hand, upregulated APOBEC is connected with high level of PD-L1 expression (Boichard et al., 2017). Proteogenomic studies have shown that APOBEC features promote an active immune microenvironment associated with PD-L1 mRNA expression, particularly in luminal breast cancer subtypes (Ohtsubo et al., 2017). Thus, APOBEC-mediated mutations may facilitate immune escape in cancer cells. Conversely, in breast cancer patients with somatic BRCA1/2 mutations, APOBEC-mediated mutations may be passenger mutations, particularly when the BRCA1/2 mutations have limited functional impact. For instance, APOBEC activity does not correlate with PARP inhibitor sensitivity, suggesting that APOBEC is not always a primary driver of tumor growth (Vidula et al., 2020).

Furthermore, Venkatesan et al. investigated when APOBEC3 expression is induced during cancer development. APOBEC3 protein expression peaks in the pre-invasive stage of breast cancer and occurs early in non-small cell lung cancer (NSCLC) evolution. This can be explained by DNA replication stress inducing APOBEC3 mutations, which drive the early onset of carcinoma *in situ* (CIN) and accelerate the deletion of tumor suppressor genes (TSGs) (Venkatesan et al., 2021).

## 3 The impact of APOBEC characteristics on clinical decision-making

### 3.1 APOBEC characteristics as predictors of treatment response

A3A induces a unique replication stress that sensitizes breast cancer cells to ataxia telangiectasia and Rad3-related kinase inhibitors (ATRi) but not to other replication inhibitors or DNA-damaging agents (Buisson et al., 2017; Buisson et al., 2019). ATR is a phosphoinositide 3-kinase-related protein kinase (Bentley et al., 1996), which responds to DNA double-strand breaks by coordinating replication stress, thereby maintaining genome integrity. Cancer cells have higher levels of replication stress and greater dependence on ATR, making ATRi a promising strategy for sensitizing cancer cells to DNA repair and replication-targeted therapies (Saldivar et al., 2017). Hyunho Kim et al. demonstrated that combining an ATR inhibitor with cisplatin yields promising results in treating muscle-invasive bladder cancer (MIBC) cells with high APOBEC3B expression (Kim et al., 2024). Enrichment of high TMB in mILCs, most of which harbor APOBEC signatures, has been identified as a significant determinant of response to ICI (Rizvi et al., 2015; Blank and Enk, 2015; Philips and Atkins, 2015). A large study of mutational processes in breast cancer suggested that APOBEC is a key driver of hypermutation in metastatic tumors. High TMB may benefit the use of ICIs in various cancers, including small cell lung cancer, where APOBEC enrichment may improve outcomes after immunotherapy (Wang et al., 2018). Preliminary evidence also suggests potential benefits of ICIs in breast cancer patients with enriched APOBEC features (Barroso-Sousa et al., 2020). Saranya Chumsri et al. reported that a case of PD-L1 and HER2 negative advanced breast cancer with high TMB and APOBEC features, previously considered immunologically cold, that benefited from long-term ICI therapy. Andrew A. Davis et al. demonstrated that APOBEC, as a potential biomarker in patients with blood TMB (bTMB), can identify patient groups likely to benefit from ICI treatment (Davis et al., 2023). In addition, a similar proportion of high TMB cases with APOBEC features was observed across distinct breast cancer subtypes (Chumsri et al., 2020). In a study using next-generation sequencing (NGS) for circulating tumor DNA (ctDNA) analysis, APOBEC was verified to be enriched in both high bTMB and low bTMB HR+/HER2− patients, further validating that APOBEC can be used as a biomarker by testing blood to predict response to ICI therapy and to decide whether to combine ICI with chemotherapy (Davis et al., 2023). Therefore, we propose that APOBEC mutation characteristics and TMB may serve as predictors of clinical outcome and response to ICI therapy in breast cancer patients, particularly in advanced breast cancer.

An alternative perspective posits that APOBEC activity fosters an immunologically hot tumor microenvironment, triggering antigen-specific adaptive immunity mediated by CD4^+^ T cells. This process enhances immune cell infiltration and slows tumor growth (Cescon et al., 2015; Smid et al., 2016; Chen et al., 2019). Therefore, DiMarco AV et al. explored the role of CD4^+^ T cells in anti-tumor immunity and their relationship with APOBEC, particularly in HER2-driven breast cancer (DiMarco et al., 2022). They proposed the idea of CTLA-4 inhibitors or CD4^+^ T cell adoptive transfer for patients with high APOBEC features and demonstrated that APOBEC enhances sensitivity to anti-CTLA-4 combined with anti-HER2 therapy (DiMarco et al., 2022). Nicola Cosgrove et al. found a higher prevalence of APOBEC-related mutational signatures in HER2-positive tumors, particularly the ER negative/HER2 positive subtype, correlating with increased immune cell infiltration and pathological complete response (pCR) (Cosgrove et al., 2023). However, this implies that tumors must exploit the immunogenicity generated by APOBEC for targeted therapy, rather than progressing due to high mutational burden. Thus, APOBEC is a double-edged sword: it promotes immunogenicity and anti-tumor immunity while also inducing genetic heterogeneity, subclonal diversity, and immune evasion. Therefore, APOBEC mutational features and mutant clonality hold promise as biomarkers for predicting immunotherapeutic response.

In HR positive/HER2 negative metastatic breast cancer, combining CDK4/6 inhibitors with endocrine therapy (ET) is an effective treatment, but drug resistance remains a significant challenge. Elevated APOBEC mutational signature S13 often correlates with genomic instability and acquired drug resistance, including RB1 inactivation, which confers resistance to CDK4/6 inhibitors like palbociclib (Park et al., 2023).

### 3.2 Drug resistance and therapeutic opportunities

During tumor progression, in addition to different mutational signatures, tumors also acquire drug resistance to conventional treatments. In a study of circulating tumor DNA (ctDNA) genomic profiles in advanced breast cancer, it has been demonstrated that subclonal mutations in HR-positive and HER2-negative breast cancers were enriched for APOBEC signatures and are continuously activated during endocrine therapy to modify PIK3CA (Law et al., 2016), generating frequent secondary hits of new mutations that upregulate PI3K signaling (Kingston et al., 2021). Given the crosstalk between ER and PI3K signaling pathways, PI3K inhibition increases ER-dependent transcription, leading to endocrine therapy resistance (Juric et al., 2015). Therefore, early combination of APOBEC or PI3K inhibitors with endocrine therapy may improve outcomes. In ER positive breast cancer, high expression of A3B was also associated with unfavorable clinical parameters (Sieuwerts et al., 2014). Subsequently, a study revealed that tumors harboring multiple PIK3CA mutations were associated with higher TMB and APOBEC mutation profiles (Sivakumar et al., 2023). The combination of Alpelisib (an orally PI3K inhibitor) and fulvestrant significantly improves progression-free survival (PFS) in patients with metastatic HR+ breast cancer, with adverse events that are manageable and well-tolerated (André et al., 2021).

Moreover, in non-small cell lung cancer (NSCLC), the commonly treatment with tyrosine kinase inhibitors (TKIs) can induce drug-tolerant persisters (DTPs) that highly express APOBEC3A, promoting genomic instability and the evolution of drug-resistant mutations. Thus, in turn, it induces the evolution of drug-resistant mutations in cancer cells (Isozaki et al., 2023).

### 3.3 APOBEC mutation characteristics and clinical outcomes

**FIGURE 3:**
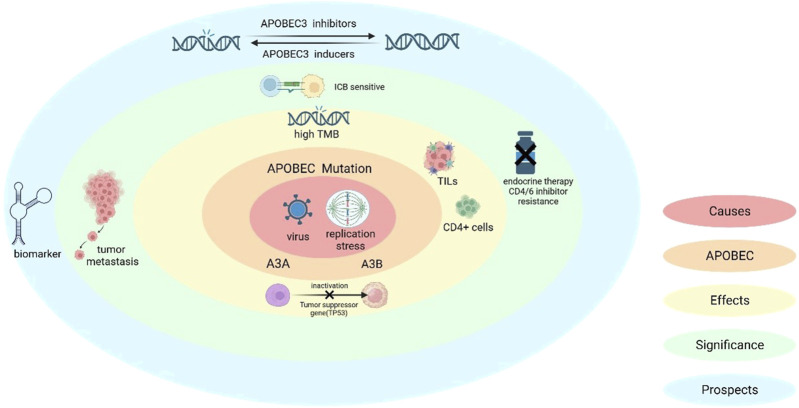
Causes of APOBEC mutations and the significance of APOBEC in breast cancer. APOBEC mutations are induced by viral infection as well as replication stress, (also A3A can induce a specific replication stress). The molecular and cellular level effects caused by APOBEC include: immune activation, which includes CD4^+^ T-cell-mediated immune processes, as well as an increase in the levels of ki-67 and TILs; in addition to inactivation of oncogenes (e.g., TP53) and high TMB production. In terms of clinical significance, high TMB makes ICB therapy sensitive, but APOBEC mutations also bring the issue of breast cancer invasion and resistance to endocrine and CDK4/6 inhibitor therapy.

APOBEC activity serves as a potential predictor of both treatment response and survival in breast cancer patients. In the HR-positive subgroup, APOBEC mutation signatures are associated with increased pathological complete response (pCR) following neoadjuvant chemotherapy (Denkert et al., 2021). However, in HR-positive and HER2-negative breast cancers, APOBEC mutational signatures correlate with poor outcomes with CDK4/6 inhibitors and endocrine therapy (Sammons et al., 2022). In addition, APOBEC is also associated with increased Ki-67 and tumor-infiltrating lymphocytes (TILs) levels, implying more aggressive tumor progression but also increased immunogenicity. This dual effect improves chemotherapy response rate (Figure 3). In conclusion, APOBEC signatures enhance chemotherapy response rates and pCR in HR-positive breast cancer but reduce the benefit of targeted therapy (Fulton-Ward and Middleton, 2023).

### 3.4 Therapeutic potential of APOBEC mutations

The correlation between APOBEC activity and high mutational load implies that APOBEC-driven mutations facilitate the disorganization of cancer cell genomes, promoting their evolution toward a more aggressive phenotype. This process is a hallmark of tumor heterogeneity. However, as previously discussed, APOBEC activity also enables tumors to thrive in an immunologically hot environment, essential for immunotherapy efficacy. This is because immunotherapy relies on the production of tumor neoantigens to elicit a robust immune response.

One study has proposed the potential for inducing APOBEC-driven mutations through first-line therapies, such as oncolytic virus therapy, and subsequently selecting cancer cells with a specific phenotype for targeted treatment. *In vitro* experiments shown that transfecting tumor cells with APOBEC expression vectors can induce APOBEC-mediated mutations, generating new epitopes that stimulate T cells. This approach, known as heteroclitic epitope-activated therapy (HEAT), leverages APOBEC-induced mutations to enhance immunogenicity and drive anti-tumor immunity. Alternatively, associating mutational signature acquisition with *in situ* tumor killing can sensitize tumors to immune checkpoint blockade therapy, a strategy referred to as hypermutagenic immunotherapy (HIT) (Vile et al., 2021).

Nevertheless, in NSCLC, TKIs have been shown to induce high APOBEC3A expression, promoting genomic instability. While this allows drug-tolerant persisters (DTPs) to survive longer and gain a survival advantage, it also highlights the complex interplay between APOBEC activity and therapeutic outcomes (Vile et al., 2021).

In summary, the clinical significance and therapeutic potential of APOBEC activity present contrasting perspectives. On one hand, APOBEC-driven mutations can enhance immunogenicity and improve immunotherapy efficacy. On the other hand, they can promote genomic instability and resistance to targeted therapies. Collectively, future research is urgently needed to develop APOBEC inhibitors and inducers that can be harnessed for medical purposes, thereby optimizing the therapeutic landscape for cancer treatment.

## 4 Discussion

The APOBEC family, particularly APOBEC3, plays a profound role in cancer development, driving mutations and contributing to high tumor mutational burden. Additionally, its relevance to the cell cycle and DNA repair pathways holds promise for therapeutic opportunities. APOBEC activity serves as a driving force for cancer evolution and heterogeneity, with most highly mutated breast cancers exhibiting both SBS2 and SBS13 signatures. In breast cancer, upregulation of A3B results in a 3-5-fold increase in mutations compared to baseline, thereby accounting for the high mutational burden. Moreover, mutations in p53 lead to upregulation of A3B expression, underscoring APOBEC’s role in breast carcinogenesis. In both luminal subtypes of breast cancer, APOBEC activity promotes immune evasion of cancer cells by increasing PD-L1 expression, thereby creating a relatively “cold” tumor immune microenvironment.

Exploring of APOBEC’s role in breast cancer development has informed strategies to enhance treatment efficacy. Immune checkpoint inhibitors (ICIs) have been shown to be effective in breast cancer patients with APOBEC-enriched profiles, based on the established relationship between APOBEC and the cell cycle. In HER2-positive breast cancer with APOBEC-enriched features, combining anti-CTLA-4 with anti-HER2 therapy may offer benefits. In patients resistant to endocrine therapy, APOBEC features suggest that PI3K or APOBEC inhibitors could improve treatment efficacy.

APOBEC acts as a double-edged sword. On one hand, it can promote immunogenicity, trigger antitumor immunity, increase immune infiltration, and slow breast cancer growth. On the other hand, it can generate genetic heterogeneity, promote subclonal diversity, and accelerate tumor progression and immune evasion. Therefore, new strategies aim to find a “balance” between these opposing effects and harness appropriate APOBEC mutations, such as heteroclitic epitope-activated therapy (HEAT) and hypermutagenic immunotherapy (HIT). The clinical value of these approaches still requires further exploration (Figure 3).

Overall, the impact of antitumor immunity is often overshadowed by the effects of drug-resistant mutations, leading to the progression and growth of cancer cells. However, it is clear that APOBEC is a valuable predictor of breast cancer treatment outcomes and has significant potential to guide clinicians in drug selection and efficacy evaluation. Additionally, APOBEC represents a promising therapeutic target for future development.

## 5 Conclusion

APOBEC enzymes epitomize the Janus-faced nature of cancer genomics—driving malignant progression while exposing therapeutic vulnerabilities. Validated as biomarkers for ICI response and PI3K inhibitor efficacy, APOBEC signatures are poised to guide precision therapy. Future success hinges on resolving key paradoxes: amplifying immunogenic mutations without fueling resistance, and selectively targeting APOBEC in metastatic niches. Integrating APOBEC biology into clinical algorithms will require multi-omics profiling and innovative clinical trial designs, ultimately transforming this molecular foe into an ally against breast cancer.
